# Concordance Between Electronic Clinical Documentation and Physicians’ Observed Behavior

**DOI:** 10.1001/jamanetworkopen.2019.11390

**Published:** 2019-09-18

**Authors:** Carl T. Berdahl, Gregory J. Moran, Owen McBride, Alexandra M. Santini, Ilya A. Verzhbinsky, David L. Schriger

**Affiliations:** 1 National Clinician Scholars Program, University of California, Los Angeles; 2 Olive View, Department of Emergency Medicine, University of California, Los Angeles; 3 Alameda Health System, Department of Emergency Medicine, Highland Hospital, Oakland, California; 4 University of California, Santa Cruz; 5 University of California, Los Angeles; 6 Department of Radiological Sciences, Stanford University, Stanford, California; 7 Department of Emergency Medicine, University of California, Los Angeles

## Abstract

**Importance:**

Following the adoption of electronic health records into a regulatory environment designed for paper records, there has been little investigation into the accuracy of physician documentation.

**Objective:**

To quantify the percentage of emergency physician documentation of the review of systems (ROS) and physical examination (PE) that observers can confirm.

**Design, Setting, and Participants:**

This case series took place at emergency departments in 2 academic medical centers between 2016 and 2018. Participants’ patient encounters were observed to compare real-time performance with clinical documentation.

**Exposures:**

Resident physicians were shadowed by trained observers for 20 encounters (10 encounters per physician per site) to obtain real-time observational data; associated electronic health record data were subsequently reviewed.

**Main Outcomes and Measures:**

Number of confirmed ROS systems (range, 0-14) divided by the number of documented ROS systems (range, 0-14), and number of confirmed PE systems (range, 0-14) divided by the number of documented PE systems (range, 0-14).

**Results:**

The final study cohort included 9 licensed emergency medicine residents who evaluated a total of 180 patients (mean [SD] age, 48.7 [20.0] years; 91 [50.5%] women). For ROS, physicians documented a median (interquartile range [IQR]) of 14 (8-14) systems, while audio recordings confirmed a median (IQR) of 5 (3-6) systems. Overall, 755 of 1961 documented ROS systems (38.5%) were confirmed by audio recording data. For PE, resident physicians documented a median (IQR) of 8 (7-9) verifiable systems, while observers confirmed a median (IQR) of 5.5 (3-6) systems. Overall, 760 of 1429 verifiable documented PE systems (53.2%) were confirmed by concurrent observation. Interrater reliability for rating of ROS and PE was more than 90% for all measures.

**Conclusions and Relevance:**

In this study of 9 licensed year emergency medicine residents, there were inconsistencies between the documentation of ROS and PE findings in the electronic health record and observational reports. These findings raise the possibility that some documentation may not accurately represent physician actions. Further studies should be undertaken to determine whether this occurrence is widespread. However, because such studies are unlikely to be performed owing to institution-level barriers that exist nationwide, payers should consider removing financial incentives to generate lengthy documentation.

## Key Points

**Question:**

How closely does documentation in electronic health records match the review of systems and physical examination performed by emergency physicians?

**Findings:**

In this case series of 9 licensed emergency physician trainees and 12 observers of 180 patient encounters, 38.5% of the review of systems groups and 53.2% of the physical examination systems documented in the electronic health record were corroborated by direct audiovisual or reviewed audio observation.

**Meaning:**

These findings raise the possibility that some physician documentation may not accurately represent actions taken, but further research is needed to assess this in more detail.

## Teaser

This case series study examines how closely documentation in electronic health records matches the review of systems and physical examination performed by emergency physicians.

## Introduction

As recently as the 1990s, the medical record’s principal purpose was to document a physician’s findings and assessment for future reference. In 1995, the Centers for Medicare & Medicaid Services (CMS) introduced policies tying reimbursement to documentation.^1,2^ Thereafter, CMS rules and regulations have shaped the content of physician documentation. Additionally, the adoption of electronic health records has made autopopulated text a prevalent mechanism for creating content.^3,4,5^

Little investigation has been done to quantify the accuracy of physician documentation.^6^ However, in 2017, CMS reported that it was working to reform documentation rules and regulations, noting “there may be unnecessary burden with these guidelines and that they are potentially outdated…. [We] believe this is especially true for the requirements for the history and the physical exam.”^7^

Certain sections of emergency physician documentation may be particularly vulnerable to faults in accuracy. While physicians may commonly dictate^8^ or type a customized history of present illness or medical decision-making note, the review of systems (ROS) and the physical examination (PE) sections of the electronic medical record may be prone to inaccuracy owing to widespread use of autopopulated text. In this study of emergency medicine resident physicians, concurrent observation, review of audio recordings of encounters, and analysis of documentation were used to quantify the accuracy of documentation of the ROS and PE in electronic health records.

## Methods

### Ethics

Institutional review boards at both participating sites approved the research protocol. (The names of the participating sites are being withheld to protect participant privacy.) To minimize the risk of social desirability bias and the Hawthorne effect on the validity of study findings, participating physicians were told at the time of initial written consent that the study was a time-motion study that aimed to understand how they performed histories and PEs. At the conclusion of the study, a 30-minute debriefing conference call was held in which participating physicians were explicitly told the intent to determine documentation accuracy. Study personnel then presented the results in departmental grand rounds, and participants were approached individually and offered another opportunity to withdraw their data after being included in a draft of the manuscript.

### Design and Setting

This was a case series study designed to quantify differences between electronic health record documentation and observed performance of the ROS and the PE by fully licensed emergency medicine resident physicians. These physicians were shadowed in 2 teaching hospital emergency departments associated with the same residency program. Data collection occurred between 2016 and 2018. This article follows the Strengthening the Reporting of Observational Studies in Epidemiology (STROBE) reporting guideline.^9^

### Participants

#### Physicians

Physicians were eligible for enrollment if they were licensed emergency medicine residents working at both study sites. Study personnel received written informed consent from physician participants at enrollment, informing them that their encounters with patients would be observed and audio recorded.

#### Patients

Patient participants were considered eligible and approached for consent if they spoke English or Spanish. They were excluded from participation if their emergency severity index^10^ at triage was 1 (most severe) or 5 (least severe) or if a medical student participated in the encounter. Patients, like physicians, were told that the encounter would be observed and audio recorded but that their care would not be affected. Research assistants observed each physician’s sequential patient encounters until 10 patient-physician encounters were observed for each physician at each site. This typically occurred during several shifts for each resident. Patients provided verbal informed consent for review of their medical records, but unlike physician participants, they were not deceived about the study’s intent.

#### Observers

The research team used 12 observers; 2 observers (17%) were practicing attending emergency physicians (C.T.B. and D.L.S.). The remaining 10 observers were undergraduate students interested in medicine who were selected as the most outstanding observers from a pool of 28 applicants. Training included 2 two-hour sessions in which observers learned about common PE maneuvers, practiced with a PE template (described later), participated in a live examination, and completed a final, video-based examination. All participating observers scored higher than 97% on the video-based examination, which was scored against a key created by 2 of us (C.T.B. and D.L.S.). During data collection, observers were instructed to closely shadow 1 physician at a time, entering rooms with the physician multiple times if necessary to audio record and collect PE data at all occasions of contact with patients. Observers shadowed a single physician for 4 to 8 hours at a time.

### Data Collection

#### Overview

We collected 5 types of data, as follows: (1) descriptive data about encounters and participants, (2) ROS observation data (in the form of an audiotape), (3) observed PE activities, documented concurrently on standardized forms, (4) ROS documentation in the electronic health record, and (5) PE documentation in the electronic health record. Audio recordings of encounters were made using 2 TASCAM DR-05 portable digital recorders (TEAC Corporation) placed in different parts of the examination room. Abstractors of medical record documentation were masked from audio and observational data from the encounter, and abstractors of audio and observational data were masked from documentation data.

#### Data Describing Patients and Visits

Information was extracted from the electronic health record denoting patient sex, age, encounter emergency severity index triage score (range, 2-4), reason for visit (as interpreted by 2 of us [C.T.B. and D.L.S.] using a combination of nurse-generated chief concern and the history of present illness), *International Statistical Classification of Diseases and Related Health Problems, Tenth Revision *(*ICD*-*10*) emergency department diagnosis, and patient disposition (ie, admission, discharge, or other). Observers also recorded duration of patient-physician history and PE, language of encounter (ie, English or Spanish), and presence or absence of a scribe. Data related to physician characteristics and evaluation and management coding were not collected.

#### Observation of ROS

Observers audio recorded patient-physician encounters. At least 1 of us (C.T.B. or D.L.S.) listened to each audio recording, using a standardized checklist to record when physicians queried specific symptoms or patients offered them. The checklist was made by combining ROS-relevant material published by CMS, common medical textbooks, and locally available templates into a single comprehensive checklist of commonly reviewed symptoms and their associated body systems (eMethods in the Supplement). If a symptom mentioned in the encounter was not listed on the checklist, the reviewer added the symptom to the encounter’s checklist under the most relevant body system. In cases where a symptom could be attributed to multiple systems, abstractors were instructed to place the symptom in the system that maximized the total number of systems reviewed. After assigning all symptoms to a body system, the number of systems queried and the identity of each system were recorded in a database. The checklist was validated by having 2 of us (C.T.B. and D.L.S.) score 10 cases independently and compare results.

#### Observation of PE

Each encounter was observed by at least 1 trained observer and often by 2 when there were multiple research assistants in the department. The observer used a standardized checklist to record the performance of discrete PE maneuvers (eMethods in the Supplement). The checklist was validated by having 2 of us (C.T.B. and D.L.S.) score 10 cases independently and compare their results. If a maneuver was not listed on the PE checklist, the observer was instructed to write it on a blank line. An algorithm was created to map each maneuver to all relevant body systems. For example, palpating the cervical spine would give physicians credit for a musculoskeletal examination and a neck examination. After assigning all maneuvers to relevant body systems, the number of systems examined and the identity of each system were recorded in a database. The list of body systems was derived from the 1995 and 1997 documentation rules and regulations published by CMS.^1,2^

#### Abstraction of ROS Documentation

At least 1 of us (C.T.B. or D.L.S.) reviewed the documentation for each case to determine how many ROS body systems the treating physician recorded as reviewed. Reviewers extracted all symptoms listed as reviewed in either the ROS or the history of present illness portions of the electronic medical record and assigned them to a body system using the same checklist as in the ROS observation measurement. In cases where a symptom could be attributed to multiple systems, reviewers were instructed to place the symptom in the system that minimized the number of systems. This was the opposite of what reviewers did when reviewing live audio because the study team wanted to give participants the benefit of the doubt overall. In other words, the intent was to maximize the count of systems performed during the encounter and to minimize the count of systems documented.

When physicians documented that all other systems were reviewed and found to be negative or an equivalent statement, this attestation was interpreted to signify that all 14 systems were documented. Encounters were excluded from ROS analysis in the 3 cases where the physician indicated that the patient could not provide appropriate answers to ROS inquiries. These cases were not excluded from PE analysis.

#### Abstraction of PE Documentation

At least 1 of us (C.T.B. or D.L.S.) reviewed PE documentation to determine how many body systems the treating physician reported examining. Reviewers first recorded the number and identity of the systems that were listed as bullet points. Second, reviewers assessed each system to determine whether the physician had recorded any verifiable content in that body system. Verifiable content was defined as any text that implied the physician performed an observable maneuver or asked the patient to follow a command. For example, the statement “lungs clear to auscultation” was considered verifiable because it implied the physician had performed auscultation with a stethoscope, an action that could be observed. In contrast, the statement “respirations unlabored” was not considered verifiable. The number and identity of systems containing at least 1 verifiable action were recorded.

The constitutional and psychiatric systems were excluded from PE analysis because documentation rarely included components that were verifiable (eg, the statement “normal affect,” with no additional documentation of the psychiatric examination). The final physician note, signed by both attending and resident physicians, was used in all analyses. Whether or not the note was prepared by a scribe, resident and attending physicians had the ability to edit ROS and PE documentation based on their findings.

### Outcome Measures

Primary outcome measures were as follows: (1) percentage of documented ROS systems confirmed by observation and (2) percentage of verifiable PE systems confirmed by observation. These outcomes were reported as percentage of confirmed documentation. The number of false-positive events (unsubstantiated documentation) and false-negative events (failure to document an ROS or PE finding) were calculated, and outcomes were stratified by physician and site.

Theoretically, some inaccuracies in documentation could have been associated with more harm than others. For example, for a patient reporting shortness of breath, documenting a lung examination that was not performed might have been associated with greater consequences than documenting a hearing examination that was not performed. To investigate the nature of documentation inaccuracies in the sample, a relevance analysis was performed in which the frequency of unsubstantiated PE documentation was cross-tabulated for 3 body regions (ie, ears, nose, and throat; cardiopulmonary; and abdomen and genitourinary) and 3 reason for visit classes (ie, ears, nose, and throat; chest pain or shortness of breath; and abdominal or genitourinary symptoms). This relevance analysis has not been validated in prior work related to documentation accuracy.

To calculate interrater reliability, data were compared from 2 of us (C.T.B. and D.L.S.) for 20 randomly selected cases for 3 data collection activities, as follows: (1) abstraction of ROS acquisition from the audio tape, (2) abstraction of ROS from the medical record, and (3) abstraction of the PE from the medical record. Additionally, the reliability of research assistants’ concurrent observation of the PE was assessed using the 53 patients whose examination was independently witnessed and recorded by 2 research assistants.

### Data Analysis

Descriptive analysis was performed using STATA version 14.2 (StataCorp). Figures were produced with STATA and MATLAB version 9.0 (MathWorks).

## Results

Initially, 12 physicians were approached to participate, and all agreed. Data were collected on 12 participating physicians in 240 encounters (10 per site per physician) for approximately 1 year between 2016 and 2018. After the element of deception was revealed to physician participants, 3 physicians elected to withdraw participation because of concerns about privacy. As a result, data from 9 physician participants were included in this report.

Encounter observations lasted a median (interquartile range [IQR]) of 6.6 (4.7-8.5) minutes. Table 1 describes characteristics of the encounters in more detail. Patients had a mean (SD) age of 48.7 (20.0) years, and 91 (50.5%) were women. Interrater reliability between independent raters was 98.2% for medical record abstraction of the ROS, 96.3% for medical record abstraction of the PE, 91.8% for ROS abstraction from the audiotape, and 96.8% for PE observation (eTable 1 in the Supplement).

**Table 1.** Characteristics of 180 Patient-Physician Encounters

Characteristic	No. (%)		
	Overall (N = 180)	Site 1 (n = 90)	Site 2 (n = 90)
Patient age, y			
<40	58 (32.2)	33 (36.7)	25 (27.8)
40-64	89 (49.4)	34 (37.8)	55 (61.1)
≥65	33 (18.3)	23 (25.6)	10 (11.1)
Patient sex			
Male	89 (49.4)	50 (55.6)	39 (43.3)
Female	91 (50.5)	40 (44.4)	51 (56.7)
Emergency severity index			
2	30 (16.7)	18 (20.0)	12 (13.3)
3	147 (81.7)	71 (78.9)	76 (84.4)
4	3 (1.7)	1 (1.1)	2 (2.2)
Admitted to hospital	47 (26.1)	29 (32.2)	18 (20.0)
Spanish language used in encounter	36 (20.0)	0	36 (40.0)
Scribe assisted with documentation	88 (48.9)	88 (97.8)	0
Reason for visit category			
Gastrointestinal	38 (21.1)	19 (21.1)	19 (21.1)
Cardiovascular	31 (17.2)	11 (12.2)	20 (22.2)
Neurologic or psychiatric	22 (12.2)	12 (13.3)	10 (11.1)
Genitourinary	16 (8.9)	4 (4.4)	12 (13.3)
Musculoskeletal	14 (7.8)	8 (8.9)	6 (6.7)
Integumentary	12 (6.7)	6 (6.7)	6 (6.7)
Respiratory	10 (5.6)	5 (5.6)	5 (5.6)
Constitutional	8 (4.4)	7 (7.8)	1 (1.1)
Ears, nose, and throat	8 (4.4)	6 (6.7)	2 (2.2)
Trauma	8 (4.4)	7 (7.8)	1 (1.1)
Eyes	2 (1.1)	1 (1.1)	1 (1.1)
Other	11 (6.1)	4 (4.4)	7 (7.8)

**Figure 1.**
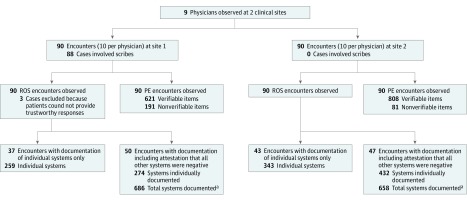
Overview of the Study PE indicates physical examination; ROS, review of systems. ^a^Under the assumption that attestation that all other systems were negative indicated that 14 systems were discussed.

Figure 1 provides an overview of observation and documentation practices for all encounters at both study sites. For the ROS analysis, 3 encounters were excluded owing to provider attestation that the patient could not provide adequate responses to ROS inquiry. Of 177 records with complete ROS documentation, physicians documented systems directly in 80 encounters (45.2%), accounting for 602 total systems, and they used the all other ROS negative statement in 97 encounters (54.8%), accounting for 1344 systems.

**Figure 2.**
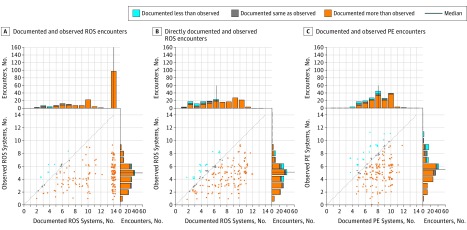
Scatterplot of the Number of Systems Documented vs the Number Observed A, The assumption was that attestation of all review of systems (ROS) are negative denoted documentation of 14 systems. B, The attestation of all ROS are negative is ignored, so systems directly documented are displayed. C, Scatterplot depicting verifiable physical examination (PE) documentation and observation. A-C, Each dot represents 1 patient-physician encounter; orange dots indicate that the number of systems documented exceeded the number observed, blue dots when the opposite occurred, and gray dots when the number of systems matched. The dotted line represents ideal physician documentation behavior, ie, when documentation matches what transpired. The histograms show the distribution of the number of systems documented and discussed, again with orange and blue depicting overdocumentation and underdocumentation and gray representing neither. The navy blue line represents the median number of systems on each histogram. Random jitter has been applied to scatterplot points to reduce superimposition of dots.

Physicians documented a median (IQR) of 14 (8-14) systems in ROS. Audio-recorded data supported a median (IQR) of 5 (3-6) systems discussed. Under the assumption that the attestation all other ROS are negative meant 14 systems were discussed, observation confirmed 755 of 1961 documented systems (38.5%). If attestation that all other ROS are negative was ignored, observation confirmed 707 of 1304 directly documented systems (54.2%) (Figure 2A and B). When physicians documented all other ROS are negative, the median (IQR) number of systems discussed was 5 (4-6). When physicians directly documented all systems, the median (IQR) number of systems discussed was 4 (3-6) (eFigure in the Supplement).

For the PE, physicians documented a total of 1701 systems in 180 encounters. Of 1701 systems documented, 272 (15.9%) contained exclusively nonverifiable content.

Concerning verifiable PE content, physicians documented a median (IQR) of 8 (7-9) systems, while observers corroborated a median (IQR) of 5.5 (3-6) (Figure 2C). Overall, observation confirmed 760 of 1429 verifiable PE systems documented (53.2%).

The study was not powered to detect differences in documentation accuracy by visit characteristics. However, we found the following: (1) the accuracy of ROS and PE documentation were 36.7% and 53.6%, respectively, for encounters with scribes vs 40.1% and 52.8% for encounters without scribes; (2) encounter language of Spanish was associated with ROS and PE accuracy of 36.6% and 52.4%, respectively, vs 39.0% and 53.4% for encounters in English; and (3) ROS and PE documentation accuracy for admitted patients were 34.9% and 55.3%, respectively, vs 40.1% and 52.4% for discharged patients (eTable 2 in the Supplement).

In the relevance analysis, the frequency of unsubstantiated PE documentation for 3 body regions (ie, ears, nose, and throat; cardiopulmonary; and abdomen and genitourinary) was cross-tabulated against 3 reasons for visit classes (ie, ears, nose, and throat; chest pain or shortness of breath; and abdominal or genitourinary symptoms). For patients presenting with gastrointestinal or genitourinary concerns, an unsubstantiated abdominal or genitourinary examination was documented in 3 of 55 instances (5.4%); for the same group of patients, an unsubstantiated ear, nose, and/or throat examination was documented in 27 of 33 (81.8%) instances (Table 2).

**Table 2.** Cross-tabulation of Frequency of Unsubstantiated Physical Examination Documentation by Reason for Visit

Reason for Visit Category	Physical Examination Region, No./Total No. (%)		
	Ears, Nose, and Throat	Cardiopulmonary	Gastrointestinal or Genitourinary
Ears, nose, and throat	0/8	2/7 (29)	3/5 (60)
Chest pain or shortness of breath	22/26 (85)	4/38 (11)	13/39 (33)
Gastrointestinal or genitourinary	27/33 (82)	8/45 (18)	3/55 (5)

**Figure 3.**
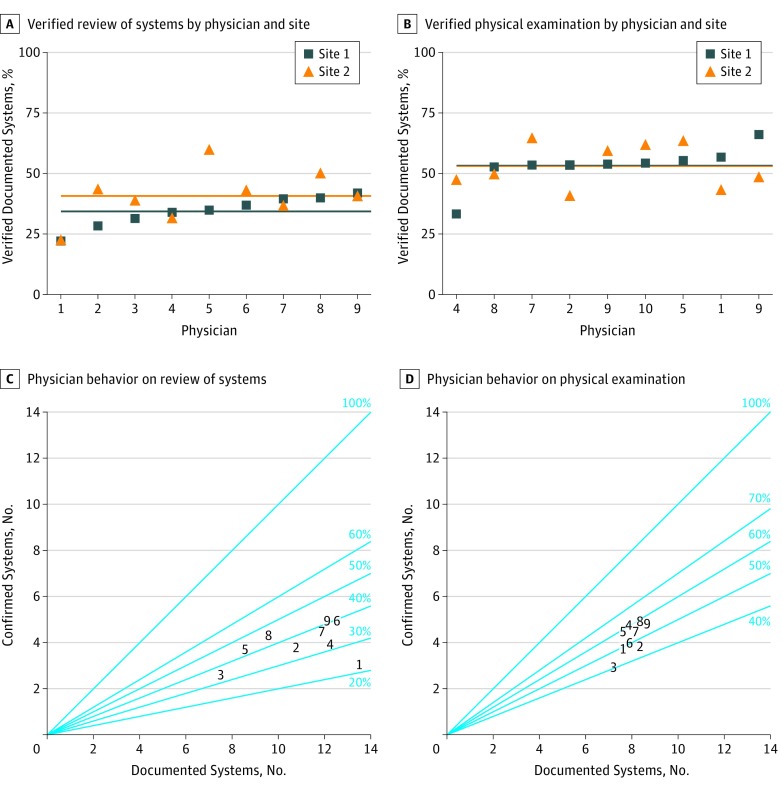
Individual Physician Behavior on Review of System and Physical Examination Documentation A-B, Each point represents the percentage of documented systems that were confirmed by observation. Each blue square represents 1 physician’s behavior at site 1, and each orange triangle represents the same physician’s behavior at site 2. Horizontal lines represent the mean performance for each physician at each site. C-D, The number of documented systems is on the x-axis, while the number of confirmed systems is on the y-axis. A perfect performance would lie on the diagonal line labeled 100%. Each physician is represented by a number (1-9), which is consistent across all 4 plots.

Figure 3 displays the accuracy of ROS and PE documentation by individual physicians and by site. It shows all participants behaved similarly at both sites.

## Discussion

This study demonstrated inconsistencies between resident physician documentation and observed behavior. Furthermore, the relevance analysis, in which the reason for visit was cross-tabulated against the percentage of unsubstantiated physical examination by body system (Table 2), demonstrated that unsubstantiated documentation is more common when it may be less clinically relevant.

Previous investigators have researched nurse documentation accuracy,^11^ compared accuracy of paper with electronic health records,^12,13^ and analyzed accuracy of physician documentation of discrete fields, such as diagnoses.^14,15^ However, to our knowledge, no other study has attempted to quantify the accuracy of electronic physician documentation using concurrent observation. The lack of literature is likely because of the challenge of data collection, the expense of concurrent observation, physician resistance to auditing,^16^ and the desire to preserve an image of physician infallibility.^17^ However, stakeholders have identified flaws in electronic health records as a serious problem. For example, in a policy position paper from the American College of Physicians, the panel reports, “the primary goal of electronic health record-generated documentation should be concise, history-rich notes that reflect the information gathered and are used to develop an impression, a diagnostic and/or treatment plan, and recommended follow-up.”^6^ Thus, payers should consider removing incentives to document lengthy ROS and PE—a change that CMS may be moving toward incrementally, given recent announcements regarding physician payment documentation requirements for outpatient office visits that will take effect in 2021.^18,19^

This study was designed to observe resident physicians at 2 clinical sites because we wanted to investigate whether site-specific billing pressures drove different intensities of documentation inaccuracy. Notwithstanding confounding factors that are discussed in the Limitations section, physicians behaved similarly at the 2 sites, which suggested that a physician’s performance at 1 institution may be associated with performance at another.

### Limitations

This study had several limitations. First, the final sample of 9 physicians was small, and all encounters occurred at sites associated with a single emergency medicine residency program, so generalizability is limited. Second, despite the fact that licensed resident physicians commonly practice emergency medicine independently at nontraining emergency department sites, their behavior may not accurately represent that of attending emergency physicians. Third, observation of physicians may have been biased for several reasons, including the inability of nonphysician observers to detect examination maneuvers that only a physician would notice, failure to capture interactions with patients that occurred when observers were not accompanying physicians, induction of changes in physician behavior owing to a Hawthorne effect, and possible introduction of selection bias owing to occasional nonsequential recruitment of patients.

Fourth, it was not possible to thoroughly investigate the influences of various factors on documentation inaccuracy, including language of encounter, use of scribes, time between encounter and completion of the note, and autopopulated text, such as templates, drop-down menus, macros, checkboxes, and copy-paste functionality. Similarly, site-specific factors were unmeasured and unaccounted for, including the severity and number of patients seen per hour, attending physician factors, and more. Fifth, consensus among the research team was used to establish a priori standards for which common phrases implied a verifiable examination maneuver. It is possible that other physicians would disagree with some of these decisions.

## Conclusions

In this study of 9 licensed emergency physicians, there were inconsistencies between documentation of ROS and PE findings in the electronic health record and reports from observers. These results raise the possibility that some documentation may not accurately represent physician actions. Further studies should be undertaken in other clinical settings to determine whether this occurrence is widespread. However, because such studies are unlikely to be performed owing to institution-level barriers that exist nationwide, payers should consider removing financial incentives to generate lengthy documentation.

## Notes

Supplement. **eMethods.** List of Systems, Checklists, and Abstraction Forms **eFigure.** Analysis of All Other ROS Negative Attestation **eTable 1.** Interrater Reliability Calculations for 4 Data Collection Mechanisms **eTable 2.** Percent of Confirmed Documentation by Visit Characteristics
