# Supplementary material for: Concordance Between Electronic Clinical Documentation and Physicians’ Observed Behavior
Source: JAMA Netw Open. 2019 Sep 18;2(9):e1911390. doi: 10.1001/jamanetworkopen.2019.11390 (PMC6751766; doi:10.1001/jamanetworkopen.2019.11390)
Supplement: Supplement. — eMethods. List of Systems, Checklists, and Abstraction Forms eFigure. Analysis of All Other ROS Negative Attestation eTable 1. Interrater Reliability Calculations for 4 Data Collection Mechanisms eTable 2. Percent of Confirmed Documentation by Visit Characteristics [file jamanetwopen-2-e1911390-s001.pdf]

# Supplementary Online Content

Berdahl CT, Moran GJ, McBride O, Santini AM, Verzhbinsky IA, Schringer DL. Concordance between electronic clinical documentation and physicians' observed behavior. *JAMA Netw Open*. 2019;2(9):e1911390. doi:10.1001/jamanetworkopen.2019.11390

**eMethods.** List of Systems, Checklists, and Abstraction Forms

**eFigure.** Analysis of All Other ROS Negative Attestation

**eTable 1.** Interrater Reliability Calculations for 4 Data Collection Mechanisms

**eTable 2.** Percent of Confirmed Documentation by Visit Characteristics

This supplementary material has been provided by the authors to give readers additional information about their work.

© 2019 Berdahl CT et al. *JAMA Network Open.*

## eMethods. List of Systems, Checklists, and Abstraction Forms

### List of Recognized ROS Systems

Constitutional

Eyes

Ears, Nose, & Throat

Cardiovascular

Respiratory

Gastrointestinal

Genitourinary

Musculoskeletal

Integumentary/Breast

Neurologic

Psychiatric

Endocrine

Hematology

Allergy/Immunology

© 2019 Berdahl CT et al. *JAMA Network Open.*

## List of Recognized PE Systems

*General/Constitutional (excluded)*

Head

Eyes

ENT

Neck

Chest wall

Respiratory

Cardiovascular

Abdominal

Genitourinary

Musculoskeletal

Extremities

Skin

Neurologic

Hematologic/Lymphatic

*Psychiatric (excluded)*

© 2019 Berdahl CT et al. *JAMA Network Open.*

# Data collection instruments

**ROS Audio (2 pages)**

**Patient_ID:** **Physician_ID:**

*****ROS Encounter recording checklist*****

**Constitutional:**
1a ☐ appetite gain
1b ☐ appetite loss
1c ☐ chills
1d ☐ diaphoresis
1e ☐ fatigue
1f ☐ fever
1g ☐ general weakness
1h ☐ malaise
1i ☐ night sweats
1j ☐ sleep habit change
1k ☐ sweating
1l ☐ weight gain
1m ☐ weight loss
other ______________

**Eyes:**
2a ☐ blurred vision
2b ☐ discharge
2c ☐ double vision
2d ☐ eye pain
2e ☐ light in eye hurts
2f ☐ photophobia
2g ☐ redness
2h ☐ tearing
2i ☐ vision loss
2j ☐ visual disturbance
other ______________

**ENT:**
3a ☐ cavities
3b ☐ congestion
3c ☐ deafness
3d ☐ dental pain
3e ☐ difficultswallowing
3f ☐ discharge from ears
3g ☐ epistaxis
3h ☐ hearing loss
3i ☐ hoarseness
3j ☐ nosebleed
3k ☐ odynophagia
3l ☐ pain in ears
3m ☐ pain w swallowing
3n ☐ rhinorrhea
3o ☐ ringing in ears
3p ☐ runny nose
3q ☐ sinus pain
3r ☐ sneezing
3s ☐ sore throat
3t ☐ sores in mouth
3u ☐ throat swelling
other ______________

**Cardiovascular:**
4a ☐ awakening suddenly with SOB
4b ☐ blue skin color
4c ☐ chest pain
4d ☐ claudication
4e ☐ fast heartbeat
4f ☐ irregular heartbeat
4g ☐ leg pain w walking
4h ☐ leg swelling
4i ☐ orthopnea
4j ☐ palpitations
4k ☐ PND
4l ☐ passing out
4m ☐ SOB w lying flat
4n ☐ SOB w walking
4o ☐ syncope
other ______________

**Respiratory:**
5a ☐ cough
5b ☐ coughing up blood
5c ☐ dyspnea
5d ☐ dyspnea on exertion
5e ☐ hemoptysis
5f ☐ SOB
5g ☐ snoring
5h ☐ sputum change
5i ☐ stridor / upper airway noises
5j ☐ TB test positive
5k ☐ wheezing
other ______________

**Gastrointestinal:**
6a ☐ abdominal pain
6b ☐ anorexia / appetite loss
6c ☐ bloating
6d ☐ blood in stool
6e ☐ bowel habit chg
6f ☐ constipation
6g ☐ diarrhea
6h ☐ difficulty swallowing
6i ☐ dysphagia
6j ☐ flatus
6k ☐ heartburn
6l ☐ hematemesis
6m ☐ hematochezia
6n ☐ hemorrhoids
6o ☐ incontinence(stl)
6p ☐ jaundice
6q ☐ melena
6r ☐ nausea
6s ☐ pain with swallowing
6t ☐ rectal pain
6u ☐ vomiting
6v ☐ vomiting blood
6w ☐ weight gain
6x ☐ weight loss
6y ☐ yellow skin
other ______________

**Genitourinary (M):**
7a ☐ blood in urine
7b ☐ flank pain
7c ☐ frequency
7d ☐ genital sore
7e ☐ hesitancy
7f ☐ impotence
7g ☐ incomplete bladder emptyting
7h ☐ incontinence (ur)
7i ☐ loss of libido
7j ☐ nighttime urination
7k ☐ pain with urination
7l ☐ penile discharge
7m ☐ scrotal swelling
7n ☐ sexual dysfunction
7o ☐ testicular pain
7p ☐ urgency
other ______________



© 2019 Berdahl CT et al. *JAMA Network Open.*

**Patient_ID:** **Physician_ID:**

*****ROS Encounter recording checklist*****

**GU/Gyn (F):**

- 8a ☐ blood in urine
- 8b ☐ dyspareunia
- 8c ☐ flank pain
- 8d ☐ frequency
- 8e ☐ genital sore
- 8f ☐ heavy bleeding
- 8g ☐ hematuria
- 8h ☐ hesitancy
- 8i ☐ hot flashes
- 8j ☐ incomplete bladder emptying
- 8k ☐ loss of libido
- 8l ☐ menorrhagia
- 8m ☐ missed menses/period
- 8n ☐ nighttime urination
- 8o ☐ nocturia
- 8p ☐ non-menstrual bleeding
- 8q ☐ painful sex
- 8r ☐ painful with urination
- 8s ☐ pelvic pain
- 8t ☐ sexual dysfunct.
- 8u ☐ urgency
- 8v ☐ urinary incont.

other ________________

**Musculoskeletal:**

- 9a ☐ arthritis
- 9b ☐ back pain
- 9c ☐ deformity
- 9d ☐ joint pain
- 9e ☐ joint swelling
- 9f ☐ muscle aches
- 9g ☐ muscle cramps
- 9h ☐ muscle weakness
- 9i ☐ myalgias
- 9j ☐ neck pain
- 9k ☐ stiffness
- 9l ☐ warmth to joint

other ________________

**Integumentary (skin, breast):**

- 10a ☐ breast discharge
- 10b ☐ breast lump/mass
- 10c ☐ breast pain
- 10d ☐ dryness
- 10e ☐ hives
- 10f ☐ flushing
- 10g ☐ infection
- 10h ☐ itching/pruritus
- 10i ☐ jaundice
- 10j ☐ laceration/cut
- 10k ☐ hair changes
- 10l ☐ nail changes
- 10m ☐ nevus / freckle
- 10n ☐ poor healing
- 10o ☐ rash
- 10p ☐ wound
- 10q ☐ yellow skin

other ________________

**Neuro:**

- 11a ☐ ataxia
- 11b ☐ balance problem
- 11c ☐ concentration prob
- 11d ☐ confusion
- 11e ☐ coordination prob
- 11f ☐ difficulty swallowing
- 11g ☐ dizziness
- 11h ☐ dysarthria
- 11i ☐ focal weakness
- 11j ☐ headaches
- 11k ☐ incontinence
- 11l ☐ memory loss
- 11m ☐ numbness
- 11n ☐ paralysis
- 11o ☐ paresthesias
- 11p ☐ passing out
- 11q ☐ seizures
- 11r ☐ speech disturbance
- 11s ☐ tremors
- 11t ☐ vertigo
- 11u ☐ vision loss
- 11v ☐ visual disturbance
- 11w ☐ syncope

other ________________

**Psychiatric**

- 12a ☐ altered mental status
- 12b ☐ depression
- 12c ☐ hallucinations
- 12d ☐ homicidal ideation
- 12e ☐ insomnia
- 12f ☐ memory loss
- 12g ☐ nightmares
- 12h ☐ nervous/anxious
- 12i ☐ paranoia
- 12j ☐ sadness
- 12k ☐ sleep disturbance
- 12l ☐ substance abuse
- 12m ☐ suicidal ideation

other ________________

**Endocrine:**

- 13a ☐ change in voice
- 13b ☐ cold intolerance
- 13c ☐ goiter/large thyroid
- 13d ☐ hair loss
- 13e ☐ heat intolerance
- 13f ☐ excessive thirst
- 13g ☐ excessive appetite
- 13h ☐ excessive urination

other ________________

**Heme/lymphatic:**

- 14a ☐ Anemia
- 14b ☐ Abnormal bleeding
- 14c ☐ Adenopathy/ swelling in glands
- 14d ☐ Excessive bruising

other ________________

**Allergy/immunology:**

- 15a ☐ environmental allergies
- 15b ☐ food allergies
- 15c ☐ HIV exposure
- 15d ☐ hives
- 15e ☐ persistent infections

other ________________



© 2019 Berdahl CT et al. *JAMA Network Open.*

## PE Observation (1 page)

**Physical examination checklist** **Site:**
**Patient ID:** **Physician ID:** **Scribe: Y / N** **ESI 2 / 3 / 4**

**Head/Eyes/Ears/Face:**

Scribe: ☐yes ☐no
Attending: ☐yes ☐no
MS: ☐yes ☐no

→ SCALP 1a☐Looked over carefully 1b☐Palpated carefully
→ LOOKED in EYES moving light in and out 2L☐Left 2R☐Right
→ LOOKED in EYES while pulling on eyelids 3L☐Left 3R☐Right
4☐Yes → MD & patient covered one eye and tested peripheral vision
5☐ tested EYE MOVEMENTS (eg tracking finger or penlight)
→LOOKED in EYES with ophthalmoscope 6L☐Left 6R☐Right
→LOOKED in EYES with slit lamp 7L☐Left 7R☐Right
8☐ LOOKED inside the MOUTH with a light
9☐ Tapped or touched teeth and inside of mouth
10☐ Assessed patient's bite
11☐ OBSERVED sticking out tongue
→ LOOKED with a speculum in EARS 12L☐Left 12R☐Right
→ tested HEARING 13L☐Left 13R☐Right → 13F☐By finger rub 13T☐ By tuning fork
14☐LOOKED with a speculum in the NOSE
15☐OBSERVED directed FACIAL EXPRESSION (ie smile, show teeth, puff cheeks, raise eyebrows)
16☐tested SENSATION of the FACE → 16F☐By finger rub 16P☐ By pin prick

**Neck:**

17☐ PALPATED the spine (back side MIDLINE)
18☐ PALPATED the sides of neck or near clavicles (for nodes) 18C☐ Checked PULSES in CAROTIDS
19☐ PALPATED the front while having the patient swallow (for thyroid)
20☐ LISTENED to neck with stethoscope (typically with patient holding breath)
21☐ Tested STRENGTH of shoulder shrug and/or head turn
→ INSPECTED the neck with the head turned to the side 22S☐Sitting or standing 22F☐ Lying down

**Chest**

23☐ PALPATED chest wall
24☐ performed a BREAST exam (OK if reported but not observed)
25☐ LISTENED to the BACK in multiple areas (min once on both sides) 25P☐PERCUSSED
26☐LISTENED to the FRONT while asking patient to take breath
27☐LISTENED to the FRONT in the area of the HEART (L of center) without asking for breath

**Abdomen/GU:**

28☐ LISTENED with the stethoscope over abdomen
29☐ had patient take DEEP BREATH while PALPATING RUQ (Murphy's sign) \ 30RU☐ 30LU☐ /
→ PALPATED abdomen →→→→→→→→→→→→→→→→→→→→→→→→→→→→→→→ | o |
31☐ PUSHED SLOWLY and then RAPIDLY RELEASED ABDOMEN (Rebound) / 30RL☐ 30LL☐ \
(REPORTED OR OBESERVED) → Male genital exam 32M☐ Vaginal exam 32V☐ Rectal exam 32D☐

**Back:**

33☐ PERCUSSED over the costovertebral angle (below ribs, above pelvis on sides of spine)
34☐ PALPATED midline structures

**Extremities:**

→INSPECTED OR PALPATED: 35U☐ upper limbs 35L☐ lower limbs 35P☐ pelvis stability
→Tested SENSATION: 36U☐ upper limbs 36L☐ lower limbs → 36F☐By finger rub 36P☐ By pin prick
→Used REFLEX Hammer: 37U☐upper limbs 37L☐ lower limbs
→PALPATED PULSES: 38DP☐ top of foot 38PT☐ inside of the ankle 38R☐ inside of the wrists

**MSK:**

JOINT MOVED BY DOCTOR (passive motion)
39☐ Neck 40☐ Back 41R☐Shoulder41L☐ 42R☐Elbow42L☐ 43R☐Wrist43L☐
44R☐Fingers44L☐ 45R☐Hip45L☐ 46R☐Knee46L☐ 47R☐Ankle47L☐ 48R☐Toes48L☐
PATIENT MOVES JOINT ON OWN OR AGAINST RESISTANCE (active motion)
49☐ Neck 50☐ Back 51R☐Shoulder51L☐ 52R☐Elbow52L☐ 53R☐Wrist53L☐
54R☐Fingers54L☐ 55R☐Hip55L☐ 56R☐Knee56L☐ 57R☐Ankle57L☐ 58R☐Toes58L☐

**Gait/balance:**

59W☐ OBSERVED walking → 59T☐ TANDEM gait ("on a tightrope") 59H☐ Toe or Heel Walk
60☐ OBSERVED patient with arms outstretched for drift
61☐ Had patient stand with eyes closed to check balance
62☐ Heel rub on shin → 63☐ Finger/nose/finger → 64☐ Rapid flipping of hands or fingers

**Overall: Additional**

65☐ MD never touched the patient
66☐ MD asked scribe to use normal exam template
67☐ Patient refused observation

**Obvious conditions (e.g. gross edema, shortness of breath, laceration):**

68☐ Activity 1) ____________________
69☐ Activity 2) ____________________
70☐ Activity 3) ____________________

© 2019 Berdahl CT et al. *JAMA Network Open.*

**ROS Electronic Health Record Abstraction (2 pages)**

**Patient_ID:** **Physician_ID:**

*****ROS Chart Abstraction Form*****

**Constitutional:**

- 1a ☐ appetite gain
- 1b ☐ appetite loss
- 1c ☐ chills
- 1d ☐ diaphoresis
- 1e ☐ fatigue
- 1f ☐ fever
- 1g ☐ general weakness
- 1h ☐ malaise
- 1i ☐ night sweats
- 1j ☐ sleep habit change
- 1k ☐ sweating
- 1l ☐ weight gain
- 1m ☐ weight loss

other ______________

**Eyes:**

- 2a ☐ blurred vision
- 2b ☐ discharge
- 2c ☐ double vision
- 2d ☐ eye pain
- 2e ☐ light in eye hurts
- 2f ☐ photophobia
- 2g ☐ redness
- 2h ☐ tearing
- 2i ☐ vision loss
- 2j ☐ visual disturbance

other ______________

**ENT:**

- 3a ☐ cavities
- 3b ☐ congestion
- 3c ☐ deafness
- 3d ☐ dental pain
- 3e ☐ difficult swallow
- 3f ☐ discharge from ears
- 3g ☐ epistaxis
- 3h ☐ hearing loss
- 3i ☐ hoarseness
- 3j ☐ nosebleed
- 3k ☐ odynophagia
- 3l ☐ pain in ears
- 3m ☐ pain w swallow
- 3n ☐ rhinorrhea
- 3o ☐ ringing in ears
- 3p ☐ runny nose
- 3q ☐ sinus pain
- 3r ☐ sneezing
- 3s ☐ sore throat
- 3t ☐ sores in mouth
- 3u ☐ throat swelling

other ______________

**Cardiovascular:**

- 4a ☐ awakening suddenly with SOB
- 4b ☐ blue skin color
- 4c ☐ chest pain
- 4d ☐ claudication
- 4e ☐ fast heartbeat
- 4f ☐ irregular heartbeat
- 4g ☐ leg pain w walking
- 4h ☐ leg swelling
- 4i ☐ orthopnea
- 4j ☐ palpitations
- 4k ☐ PND
- 4l ☐ passing out
- 4m ☐ SOB w lying flat
- 4n ☐ SOB w walking
- 4o ☐ syncope

other ______________

**Respiratory:**

- 5a ☐ cough
- 5b ☐ coughing up blood
- 5c ☐ dyspnea
- 5d ☐ dyspnea on exertion
- 5e ☐ hemoptysis
- 5f ☐ SOB
- 5g ☐ snoring
- 5h ☐ sputum change
- 5i ☐ stridor / upper airway noises
- 5j ☐ TB test positive
- 5k ☐ wheezing

other ______________

**Gastrointestinal:**

- 6a ☐ abdominal pain
- 6b ☐ anorexia / appetite loss
- 6c ☐ bloating
- 6d ☐ blood in stool
- 6e ☐ bowel habit chg
- 6f ☐ constipation
- 6g ☐ diarrhea
- 6h ☐ difficulty swallowing
- 6i ☐ dysphagia
- 6j ☐ flatus
- 6k ☐ heartburn
- 6l ☐ hematemesis
- 6m ☐ hematochezia
- 6n ☐ hemorrhoids
- 6o ☐ incontinence(stl)
- 6p ☐ jaundice
- 6q ☐ melena
- 6r ☐ nausea
- 6s ☐ pain with swallowing
- 6t ☐ rectal pain
- 6u ☐ vomiting
- 6v ☐ vomiting blood
- 6w ☐ weight gain
- 6x ☐ weight loss
- 6y ☐ yellow skin

other ______________

**Genitourinary (M):**

- 7a ☐ blood in urine
- 7b ☐ flank pain
- 7c ☐ frequency
- 7d ☐ genital sore
- 7e ☐ hesitancy
- 7f ☐ impotence
- 7g ☐ incomplete bladder emptyting
- 7h ☐ incontinence (ur)
- 7i ☐ loss of libido
- 7j ☐ nighttime urination
- 7k ☐ pain with urination
- 7l ☐ penile discharge
- 7m ☐ scrotal swelling
- 7n ☐ sexual dysfunction
- 7o ☐ testicular pain
- 7p ☐ urgency

other ______________



© 2019 Berdahl CT et al. *JAMA Network Open.*

**Patient_ID:** **Physician_ID:**

*****ROS Chart Abstraction Form*****

**GU/Gyn (F):**

8a ☐ blood in urine
8b ☐ dyspareunia
8c ☐ flank pain
8d ☐ frequency
8e ☐ genital sore
8f ☐ heavy bleeding
8g ☐ hematuria
8h ☐ hesitancy
8i ☐ hot flashes
8j ☐ incomplete bladder emptying
8k ☐ loss of libido
8l ☐ menorrhagia
8m ☐ missed menses/period
8n ☐ nighttime urination
8o ☐ nocturia
8p ☐ non-menstrual bleeding
8q ☐ painful sex
8r ☐ painful with urination
8s ☐ pelvic pain
8t ☐ sexual dysfunct.
8u ☐ urgency
8v ☐ urinary incont.
other ________________

**Musculoskeletal:**

9a ☐ arthritis
9b ☐ back pain
9c ☐ deformity
9d ☐ joint pain
9e ☐ joint swelling
9f ☐ muscle aches
9g ☐ muscle cramps
9h ☐ muscle weakness
9i ☐ myalgias
9j ☐ neck pain
9k ☐ stiffness
9l ☐ warmth to joint
other ________________

**Integumentary(skin, breast):**

10a ☐ breast discharge
10b ☐ breast lump/mass
10c ☐ breast pain
10d ☐ dryness
10e ☐ hives
10f ☐ flushing
10g ☐ infection
10h ☐ itching/pruritus
10i ☐ jaundice
10j ☐ laceration/cut
10k ☐ hair changes
10l ☐ nail changes
10m ☐ nevus / freckle
10n ☐ poor healing
10o ☐ rash
10p ☐ wound
10q ☐ yellow skin
other ________________

**Neuro:**

11a ☐ ataxia
11b ☐ balance problem
11c ☐ concentration prob
11d ☐ confusion
11e ☐ coordination prob
11f ☐ difficulty swallowing
11g ☐ dizziness
11h ☐ dysarthria
11i ☐ focal weakness
11j ☐ headaches
11k ☐ incontinence
11l ☐ memory loss
11m ☐ numbness
11n ☐ paralysis
11o ☐ paresthesias
11p ☐ passing out
11q ☐ seizures
11r ☐ speech disturbance
11s ☐ tremors
11t ☐ vertigo
11u ☐ vision loss
11v ☐ visual disturbance
11w ☐ syncope
other ________________

**Psychiatric**

12a ☐ altered mental status
12b ☐ depression
12c ☐ hallucinations
12d ☐ homicidal ideation
12e ☐ insomnia
12f ☐ memory loss
12g ☐ nightmares
12h ☐ nervous/anxious
12i ☐ paranoia
12j ☐ sadness
12k ☐ sleep disturbance
12l ☐ substance abuse
12m ☐ suicidal ideation
other ________________

**Endocrine:**

13a ☐ change in voice
13b ☐ cold intolerance
13c ☐ goiter/large thyroid
13d ☐ hair loss
13e ☐ heat intolerance
13f ☐ excessive thirst
13g ☐ excessive appetite
13h ☐ excessive urination
other ________________

**Heme/lymphatic:**

14a ☐ Anemia
14b ☐ Abnormal bleeding
14c ☐ Adenopathy/ swelling in glands
14d ☐ Excessive bruising
other ________________

**Allergy/immunology:**

15a ☐ environmental allergies
15b ☐ food allergies
15c ☐ HIV exposure
15d ☐ hives
15e ☐ persistent infections
other ________________



© 2019 Berdahl CT et al. *JAMA Network Open.*

## PE Electronic Health Record Abstraction (1 page)

Instructions to reviewer:

Please note whether the physician documented a verifiable exam, a non-verifiable exam, or no exam for each physical exam system. Specific exam maneuvers flagged with * should always be marked as verifiable.

| | | Verifiable | Non-verifiable | No documentation |
|---|---|---|---|---|
| **GENERAL and/or CONSTITUTIONAL** | | | | |
| **HEAD and/or FACE** | | | | |
| **EYES** | | | | |
| *Eyes - PERRL | | | | |
| *Eyes - EOMI | | | | |
| **ENT** | | | | |
| *ENT - Tympanic membranes | | | | |
| *ENT - Mucous membranes | | | | |
| **NECK** | | | | |
| **RESPIRATORY** | | | | |
| *Resp - used stethoscope | | | | |
| **CARDIOVASCULAR** | | | | |
| *CV - used stethoscope | | | | |
| *CV - pulses (look at extremity exam also) | | | | |
| **CHEST WALL and/or BREAST** | | | | |
| **ABDOMINAL incl RECTAL** | | | | |
| *GI/Abd - touched abdomen (e.g. tenderness) | | | | |
| *GI/Abd - used stethoscope | | | | |
| **GENITOURINARY** | | | | |
| **MUSCULOSKELETAL** | | | | |
| **EXTREMITIES** | | | | |
| **SKIN** | | | | |
| **NEURO** | | | | |
| *Neuro - documented intact (incl II-XII) | | | | |
| **PSYCH** | | | | |
| **HEME/LYMPHATIC** | | | | |

© 2019 Berdahl CT et al. *JAMA Network Open.*

**eFigure. Analysis of All Other ROS Negative Attestation**

Histogram of the number ROS systems discussed when physicians attested “all other ROS negative” versus without attestation. Horizontal lines depict the median number of systems discussed in each group.

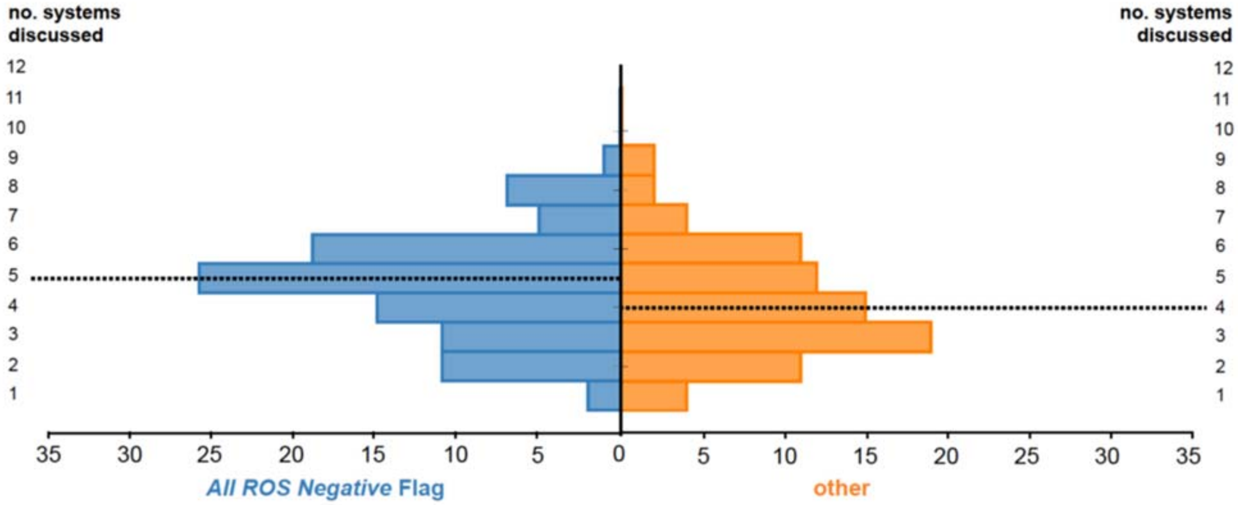


© 2019 Berdahl CT et al. *JAMA Network Open.*

**eTable 1.** Interrater Reliability Calculations for 4 Data Collection Mechanisms

| | **ROS Medical Record** | **ROS Audio** | **PE Medical Record** | **PE Observation** |
|---|---|---|---|---|
| **Number of cases used in IRR assessment** | 20 | 20 | 20 | 53 |
| **IRR case selection method** | random | random | random | when two research assistants were available to independently observe encounter and record data |
| **IRR calculation technique** | n/N (%) | n/N (%) | n/N (%) | n/N (%) |
| **1. Denominator was determined by the number of systems included in the rating tool** | 275/280 (98.2) | 257/280 (91.8) | 289/300 (98.2) | 667/689 (96.8) |
| **2. Denominator was determined by the number of occasions at least one rater noted a positive finding** (negative-negative agreement not counted) | 132/137 (96.4) | 67/90 (74.4) | 200/211 (96.4) | 221/243 (90.9) |
| **3. Denominator was determined by the number of Individual physical examination *maneuvers* (rather than number of systems) on the checklist** (negative-negative agreement not counted) | N/A | N/A | N/A | 205/260 (79) |

IRR = interrater reliability

ROS = review of systems

PE = physical examination

© 2019 Berdahl CT et al. *JAMA Network Open.*

## eTable 2. Percent of Confirmed Documentation by Visit Characteristics

| | Accuracy of documentation | |
|---|---|---|
| | **Review of Systems** n/N (%) | **Physical Examination** n/N (%) |
| **Visit Characteristics** | | |
| **Scribe** | | |
| No | 413/1029 (40.1) | 436/825 (52.8) |
| Yes | 342/932 (36.7) | 324/604 (53.6) |
| **Language of Encounter** | | |
| English | 602/1543 (39.0) | 588/1101 (53.4) |
| Spanish | 153/418 (36.6) | 172/328 (52.4) |
| **Disposition** | | |
| Discharge | 548/1368 (40.1) | 551/1051 (52.4) |
| Admission | 207/593 (34.9) | 209/378 (55.3) |

© 2019 Berdahl CT et al. *JAMA Network Open.*
